# Management of Deep Neck Infection Associated with Descending Necrotizing Mediastinitis: A Scoping Review

**DOI:** 10.3390/medicina61020325

**Published:** 2025-02-12

**Authors:** Bogdan Mihail Cobzeanu, Liliana Moisii, Octavian Dragos Palade, Mihai Ciofu, Florentina Severin, Mihai Dumitru, Luminita Radulescu, Cristian Martu, Mihail Dan Cobzeanu, Geanina Bandol

**Affiliations:** 1 ENT Clinic Department, University of Medicine and Pharmacy “Grigore T. Popa”, 700115 Iasi, Romania; 2 ENT Clinic, Clinical Rehabilitation Hospital, 700661 Iasi, Romania; 3 Emergency Clinical Hospital “Sf. Spiridon” Iasi, 700111 Iasi, Romania; 4 ENT Department, “Carol Davila” University of Medicine and Pharmacy, 011172 Bucharest, Romania; orldumitrumihai@yahoo.com; 5 Regional Oncology Institute, 700483 Iasi, Romania

**Keywords:** deep neck infection associated with descending necrotizing mediastinitis, diagnosis, multidisciplinary treatment, medical and surgical treatment

## Abstract

Deep neck infection is a pathology at the border of two specialties, otorhinolaryngology and maxillofacial surgery, and represents a medico-surgical emergency. In terms of its evolution, it can extend to the level of the thorax and result in mediastinitis, with difficult evolution and poor prognosis. The aims of this scoping review are to present the etiology, bacteriology, clinical manifestations, and diagnostics, as well as treatment, in light of the research published in the last 5 years on deep neck infection associated with descending necrotizing mediastinitis. The most common primary sources of deep neck infection are odontogenic and tonsillar. The other sources that are involved in deep neck infection are salivary glands, foreign bodies, malignancies, and iatrogenic causes after endoscopic maneuvers. The bacteriologic aspect is polymorphic, including both aerobic and anaerobic species. Complications that may appear include jugular vein thrombosis, airway obstruction, acute respiratory distress syndrome, sepsis, and disseminated intravascular coagulation. Timely diagnosis is important for ensuring the positive evolution of a deep neck infection. A CT scan is important for characterizing the nature of a deep neck lesion and identifying the spaces involved, and this method represents the gold standard for diagnosis of these lesions. Following the establishment of a definitive diagnosis, antibiotic therapy is initiated empirically, and is modified according to bacteriological exam results. The administration of antibiotics is an essential part of the treatment strategy for patients with a deep neck infection. Based on CT results, different surgical methods are applied under general anesthesia. The surgical strategy involves opening and draining the cervical spaces and debriding the necrotic tissue. In the cases of odontogenic causes, drainage and extraction of the infected teeth are performed. It is especially important to follow up on the dynamic progression of the patient. In the management of a deep neck infection associated with descending necrotizing mediastinitis, a multidisciplinary team is necessary.

## 1. Introduction

**Figure 1:**
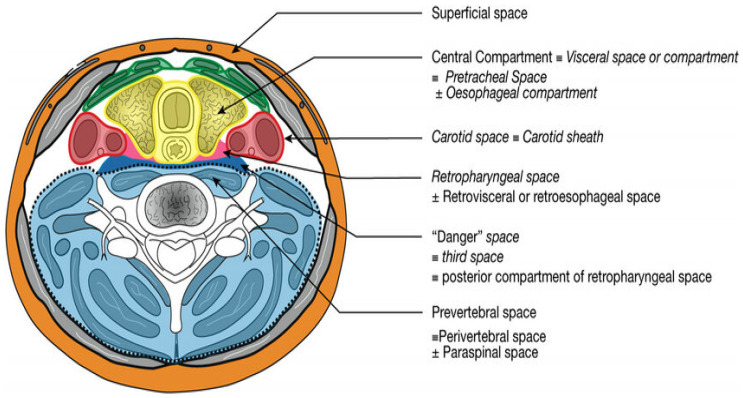
Source: The deep spaces in the neck (infrahyoid transverse section adapted from McMinn RMH, *Last’s Anatomy*) [7].

Bacterial infections and abscesses are frequently occurring diseases in the head and neck. Deep neck infection (DNI) can be caused by odontogenic infection, pharyngitis, salivary gland infection, trauma, or lodged foreign bodies. Deep neck abscesses have a fulminant evolution. The respiratory tract is compromised at an early stage, followed by rapid extension of the infection toward the mediastinum. The stages of a diagnostic (clinical and imaging) and therapeutic algorithm are essential. The neck has two states of cervical fascia that cover its contents and form the potential spaces of the head and neck. These fascial planes constitute important anatomical limitations to the spread of an infection, as well as to its targeting once their natural resistance to this spread is overcome. Therefore, anatomical considerations are essential in the treatment of deep cervical suppurations, especially when planning treatment strategies and guarding against potential complications [1]. The neck contains several cavities and fascial recesses, including the retropharyngeal, peritonsillar, masseteric, pterygopalatine maxillary, parapharyngeal, and submandibular spaces, all of which are interconnected and filled with soft connective tissue, adipose tissue, muscles, blood vessels, and nerves [2]. DNIs can spread to several of these tissue sites along interconnected soft tissues, potentially resulting in severe symptoms and poor health outcomes [2] (Figure 1).

The spread of the infection is limited by the fascial planes, but it is also directed by them when their natural resistance is exceeded. The superficial cervical fascia includes the superficial musculoaponeurotic system, facial muscles, platysma muscle, superficial blood vessels (external jugular vein), and adipose tissue. There are three important deep spaces between the layers of the deep fascia. The shape of the parapharyngeal space resembles an inverted pyramid, with boundaries between the petrous apex at the base of the skull and the greater horn of the hyoid bone. It is delimited medially by the buccopharyngeal fascia, laterally by the superficial layer of the deep cervical fascia, posteriorly by the prevertebral fascia, and anteriorly by the pterygomandibular raphe and the medial pterygoid muscle.

The cervical space is segmented by superficial and deep cervical fascia. The alar fascia extending from the base of the skull to the T2 vertebra is positioned between the prevertebral fascia and retropharyngeal space. The danger space lies behind the alar fascia and stretches down to the diaphragm [1]. Due to their close anatomical relationship, an infection in the cervical space can spread to the mediastinum. There are three pathways through which an abscess can spread along the fascial planes to the mediastinum: the pretracheal route, leading to the anterior mediastinum; the lateral pharyngeal route, leading to the middle mediastinum; and the retropharyngeal route, leading to the posterior mediastinum [3]. The danger space is named so because of the potential for the rapid spread of infection to the posterior mediastinum through the lax areolar tissue. It is a potential space between the alar and the prevertebral fasciae of the deep layer of the deep cervical fascia [3].

The “danger space” extends from the base of the skull to the diaphragm [4]. It is bounded anteriorly by the alar fascia, posteriorly by the prevertebral fascia, and laterally by the transverse processes [4]. The danger space lies posterior to the retropharyngeal space and anterior to the perivertebral space [4]. Infections in this space result indirectly from the spread of retropharyngeal, parapharyngeal, and prevertebral abscesses [4].

The danger space also contains fat. The “danger” is the tendency for infections to spread inferiorly into the mediastinum and thorax, because the loose areolar contents offer little resistance, leading to complications such as mediastinitis, empyema, and sepsis. Danger space infections initially present in the same manner as retropharyngeal infections, and a computed tomography scan is necessary in order to differentiate them [4,5,6].

In Figure 1, the orange color represents the superficial space, the yellow color indicates the pretracheal and esophageal space, the red color denotes the carotid space, the pink color indicates the retropharyngeal space, the blue color denotes the danger space, and the blue color represents the prevertebral space.

**Figure 2:**
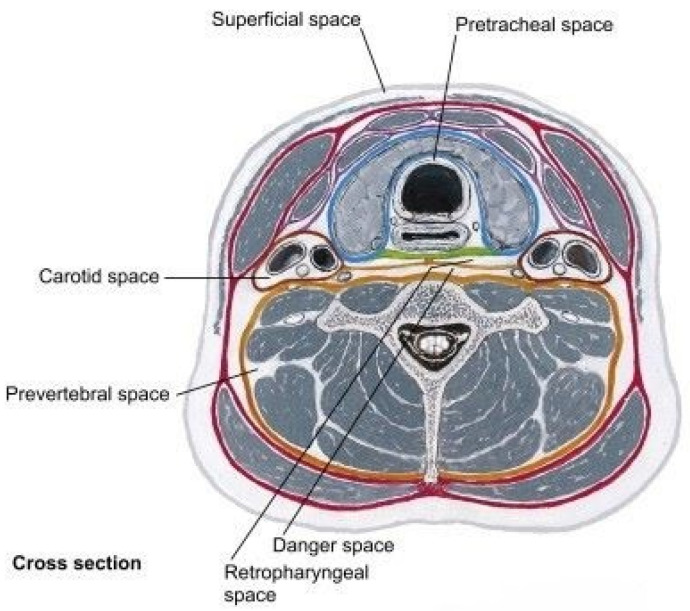
Source: Deep space neck infection: danger spaces [8].

Figure 2 shows the deep neck spaces—the pretracheal space, retropharyngeal space, prevertebral space, danger space, and carotid space, and the surrounding anatomy [8].

The red line represents the superficial cervical fascia, the blue line denotes the superficial layer of the deep cervical fascia, the green line indicates the middle layer of the deep cervical fascia, and the brown line represents the deep layer of the cervical fascia with the alar and prevertebral fasciae (sheets).

The cervicofacial region is frequently affected by suppurative processes. The anatomoclinical forms are acute cellulitis, abscess, and phlegmon. Cellulite is an expression of inflammation, practically in its pre-suppurative stage. It is characterized by vasodilatation and increased vascular permeability. Clinical examination shows swelling that is sensitive to palpation, but without areas of fluctuation. An abscess is an expression of limited suppuration, with clinical examination marking the appearance of fluid. The overlying integuments are distended and erythematous, and the general condition becomes altered, with fever and tachycardia. Phlegmon has the clinical characteristics of an abscess, but expresses extensive suppuration, without clear boundaries in relation to neighboring tissues. A massive swelling of the neck is detected during clinical examination, that is hard or fluctuant to palpation and has the presence of crepitations. In DNI, the most common symptoms are odynophagia, dysphagia, trismus, and drooling. Persistent physical signs are fever, neck adenopathy, erythema, and neck stiffness. The progression can be extremely fast, and strong suspicion of a poor prognosis is justified.

The management of DNI associated with descending necrotizing mediastinitis (DNM) is aggressive, and is immediately instituted after establishing the diagnosis. Surgical treatment (drainage and debridement), together with initially empiric antibiotic therapy, which covers aerobic and anaerobic bacteria, stabilizes cases of infection. Occasionally, only injectable antibiotic treatment is sufficient in patients who present only cellulitis or a small abscess [9,10,11]. However, in most situations, antibiotics are replaced according to the result of the culture, and in complicated cases, surgical reintervention is necessary. The mortality rate in cases of DNI with complications can increase by up to 9.3% [12].

## 2. Materials and Method

**Figure 3:**
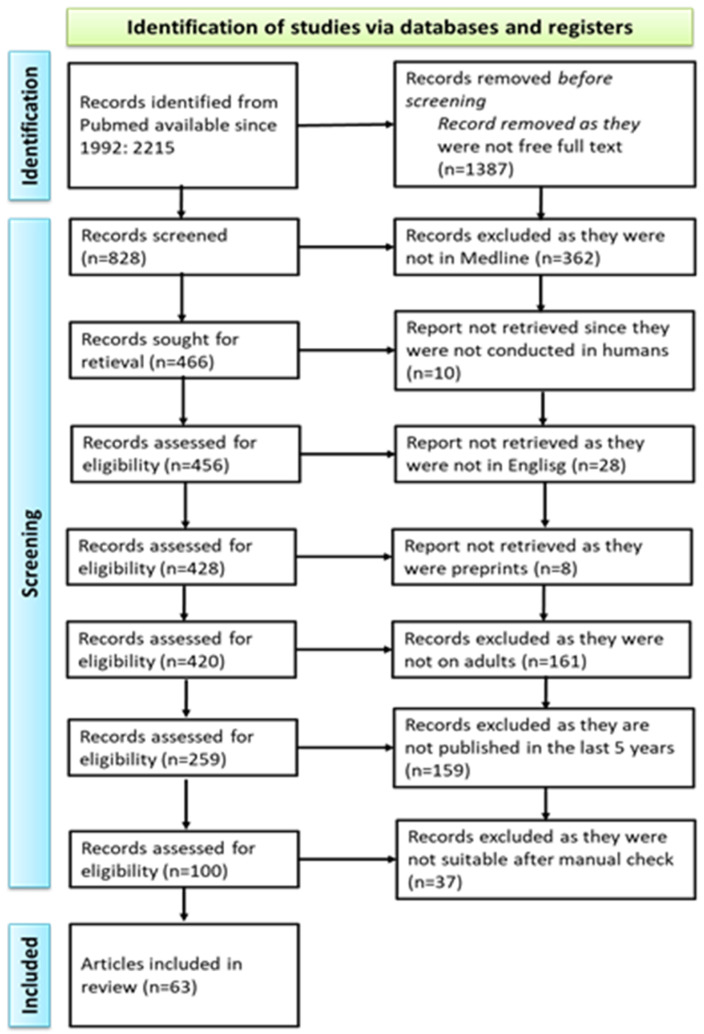
A PRISMA diagram of the current scoping review.

We queried the PubMed database using the following keywords: deep neck infection. We obtained 2215 manuscripts from the last 30 years. Further restriction of the research to articles where the free full text was available limited the number of manuscripts to 828. A total of 466 of these articles were in the Medline database. Of these, 456 manuscripts were on human subjects, of which 428 were written in English. We also excluded preprints and obtained 420 articles. Focusing only on adult patients, we obtained 259 results. Further limiting the research to the last 5 years, we obtained 100 articles, as shown below in Figure 3.

The steps followed in designing this scoping review were as follows: (1) we identified the research question, (2) we identified relevant studies, (3) we selected studies using an iterative team approach, (4) we charted the data, incorporating a numerical summary and a qualitative thematic analysis, and (5) we summarized and reported the results.

The PubMed search results outlined in Figure 3 were imported into one online cloud database. Subsequently, two groups of two reviewers (B.M.C. and O.D.P.; L.M. and M.C.) independently screened titles, abstracts, and full texts for inclusion. All discrepancies between reviewers were resolved by another set of two reviewers (F.S. and G.B.). This type of reviewer distribution and control was employed to limit possible bias. The final number of studies in the present scoping review was 74, and these studies are also included in the reference list.

## 3. Etiopathology

The most common etiological factors associated with DNI are dental infection, pharynx infection followed by upper respiratory tract infection (URTI), external or internal trauma, congenital sinus, and malignancy. Table 1 shows that in different studies from the last 15 years, the most common etiology in DNI with DNM is pharyngo-tonsillar, in contrast to DNI in which odontogenic etiology predominates. A significant percentage also represents unknown etiology, or etiology grouped under other causes [12,13,14,15,16,17,18,19].

Thus, there has been a decrease in suppurations due to odontogenic causes in the last 5 years; this is possibly also a result of the application of preventive measures in the dental network, and of the timely resolution of infections due to odontogenic causes.

In the same context, there has been an increase in DNI of unknown etiology, from 11.8% to 23.8% in the last 5 years (Table 1).

The most common spaces involved are the suprahyoid spaces. The most frequent space involved both unilaterally and bilaterally is the submandibular space (66.6%). This is followed by the sublingual and parotid spaces and the anterior visceral space.

The exact most common location of DNI remains unclear, due to varying eligibility criteria in different studies. Some authors report a high incidence of peritonsillar abscesses, though these are generally not considered deep-space infections. The submandibular space is the most frequently identified site of DNI, followed by the parapharyngeal space. Certain studies suggest that the involvement of multiple spaces in DNI may be a prognostic factor for increased complication rates, though it does not necessarily correlate with extended hospitalization [9,20,21]. According to a multivariate analysis by Staffieri et al., a leukocyte count exceeding 11,109/L is a significant predictor of extended hospitalization, though it does not imply an increased risk of complications [22].

Importantly, most lesions have an otorhinolaryngological (ORL) and oromaxillofacial (OMF) starting point. The infection spreads, through contiguity, through the lymphatic or hematogenous route, following contamination of mucosa mainly of dental, buccal, pharyngeal, laryngeal, submaxillary, or parotid glandular inflammatory origin, but also of iatrogenic origin, following endoscopic maneuvers or orotracheal intubations. Etiologically, infection is most frequently caused by aerobic and anaerobic germs. Commensal bacteria of the oropharyngeal flora play a role in the etiopathogenesis of cervical suppuration. When a bacterial or viral infection causes a physiological imbalance, these commensal bacteria become aggressive and invasive.

Depending on the virulence of the germs, but also on the characteristics of the patient (immunocompromised—cancer, HIV, diabetes mellitus, elderly) and treatment with NSAIDs or corticosteroids, the infection may remain localized for a long time, and then turn into an abscess or progress to a diffuse and extensive infection, which is sometimes necrotic, in the form of necrotic extensive cervical cellulitis or adenoflegmon [23].

Neck abscesses are produced by bacteria found in the pharynx and oral cavity. The most common bacteria involved in DNI in many studies are Streptococcus viridans and Staphylococcus aureus. In cases of DNI with DNM but also necrotizing fasciitis, Gram-negative bacteria, but also polymicrobial infections, have been described [17,23,24]. In some studies, an increased proportion of patients whose bacterial culture is negative has been found [25,26], this being due to the empiric administration of antibiotics before hospitalization.

Pyogenic streptococcus is the most aggressive form of the bacteria, because it releases streptodornases and streptokinases that determine bacterial tissue progression, as well as tissue necrosis. Anaerobic proliferation is favored by edema and venous thrombosis, which accentuate the effect of tissue anaerobic bacteria. The systemic disease thought to be most commonly associated with deep pharyngeal infections is diabetes mellitus. While Klebsiella is more typical in patients with diabetes, *Streptococci* are the most frequently isolated pathogens in non-diabetic groups [27,28]. Malnutrition and poor socioeconomic status are also considered negative predictors of complications.

As the infection progresses, local ischemia and necrosis occur. Clinical evaluation findings report palpation, the presence of crepitations or emphysema at the upper cervicothoracic level, and finally, pallor and necrosis of the skin. The main associated complication is the downward extension of the infection to the mediastinum. Necrotizing cervical fasciitis is a severe and fulminant infection [29].

Kim et al. established an algorithm that helps to identify necrotizing fasciitis in the early stages and facilitates proper surgical management [30]. They proposed six predictive factors, in the form of risk indicators (C-reactive protein, leukocytes, hemoglobin, Na, creatinine, and blood glucose) associated with CT evaluation (thickened fasciae, presence of gas, presence of small abscesses), that can be quickly applied in clinical evaluations (given the simplicity of their calculation) [30].

Necrotizing fasciitis is the most feared form of cervicomediastinal suppurations, with fulminant onset due to infection with anaerobic bacteria. Patients are severely affected, with signs of sepsis. Clinical features do not correspond to the moment of pathogenesis; the onset of symptoms occurs when there is already extensive infection. The patient may look deceptively well at first, with the rate of necrosis being disproportionate to the signs and symptoms of the infection. If early surgical treatment is not initiated, its evolution leads to death [31]. The occurrence of descending necrotizing mediastinitis is marked by the direct extension of the oropharyngeal or cervical infection to the mediastinum. The relationships between the compartments of the mediastinum are of great clinical importance, because a lesion that occupies one of the spaces (superior, anterior, middle, or posterior mediastinum) can affect neighboring structures. The most common etiology of necrotizing descending mediastinitis is pharyngeal infections, which exceed odontogenic infections in percentage. They are usually polymicrobial infections with gas-producing bacteria, which rapidly produce tissue necrosis and death by shock in patients [32,33].

Descending necrotizing mediastinitis is a complication of DNI with a frequency of 3% and a mortality rate of up to 50% [28,30,34]. The main causes of mortality are a delay in diagnosis, insufficient drainage, and debridement of the infection [30,34]. The establishment of a strong suspicion of descending necrotizing mediastinitis should be made in patients with a general condition that is highly influenced by septic shock and dyspnea. Aggressive surgical treatment to remove the necrotic tissue leads to a decrease in mortality rate and a good prognosis [35].

## 4. Clinical and Paraclinical Diagnosis

Most studies of deep throat infections in the past decade have recognized neck pain and swelling as the most prevalent symptoms [35,36,37]. In other studies, odynophagia and fever [38] were observed more frequently. Other symptoms were specific to the affected space, and included dysphagia, trismus, dysphonia, otalgia, and dyspnea [39]. Depending on the etiology of the suppurations, the first symptoms to appear worsen as the infectious process covers several cervical spaces.

The location of the infection in the pharyngo-laryngeal area can cause the appearance of dysphonia and dyspnea, with deterioration in the patient’s general condition within the first hours of the infectious process. Swelling and infiltration of the deep tissues of the neck, or extension in the submandibular area in the vicinity of the sterno-cleido-mastoid muscle, stiffen the cervical region. The patient has limited mobilization of the neck, and the tissues become infiltrative, with increased consistency and hard and sometimes erythematous surfaces. In the case of immunosuppressed patients, fever may be absent, with the clinical picture of these patients being diminished, in contrast to the case of deep tissue infection.

In cases of cervicomediastinal suppuration with late diagnosis, patients can present severe complications with aggravated symptoms, such as inspiratory dyspnea, spontaneous fistulization in the upper or external aerodigestive tract at the level of the skin, and the appearance of mediastinitis symptoms. If the infection spreads to the mediastinum, there is swelling of the base cervical region and tegumentary erythema in the cervicothoracic cape, crepitations due to gas accumulation accompanied by fluctuation, and respiratory disorders that can evolve into toxic septic shock. The general condition of the patient is greatly influenced, with low values of oxygen saturation, the presence of tachycardia, arterial hypertension, hypotension, and profuse sweating.

The clinical picture of patients with cervical suppuration, in which the extension of the infectious process includes several fascial spaces, contiguity, and infection of the anterior or posterior mediastinum, includes the appearance of subcutaneous emphysema due to anaerobic germs or visceral continuity solutions, symptoms of pneumonia with severe dyspnea, cardiac disorders, and symptoms of acute renal failure up to anuria. The risk of death for these patients is extremely high if aggressive therapeutic intervention is not undertaken in the first few hours after their presentation [35,36,37,38,39].

Physical examination consists of evaluation of the cervical region—inspection and palpation can specify the presence of erythema, fluctuance, tenderness, and the extent and location of edema; buccopharyngoscopy—inspection of the gingival mucosa, teeth, and floor of the mouth, and assessment of the palatine veil, tonsillar lobes, oropharyngeal posterior wall, and presence or absence of trismus; and assessment of the airways—laryngoscopy with flexible optical fibers for the assessment of the patency of the upper aerodigestive tracts. Upon inspection and palpation of the anterior cervicofacial and thoracic region, asymmetry of the neck can be highlighted by unilateral or bilateral subangulo-mandibular swelling, or latero-cervical or basi-cervical swelling, that descends towards the presternal region, as well as infiltrative erythematous skin that is painful on palpation, sometimes with fluctuation. Gaseous crackles can sometimes be palpated in cases of infection with anaerobic germs. Trismus occasionally appears when the infectious process invades the intermaxillary space (retromolar trigone), which makes examination difficult. In these conditions, the intermaxillary distance must be measured in order to establish the anesthetic plan for surgical treatment. Nasopharyngo-laryngeal videofibroscopic examination is an indispensable evaluation for the visualization of the upper aerodigestive tract, highlighting inflammation of the pharyngo-laryngeal mucosa, narrowing of the airways due to swelling of the epiglottis, edema of the laryngeal crown with narrowing of the glottic space, and the appearance of dyspnea. Important salivary stasis can be found in the pyriform sinuses and the bulging of the back wall of the pharynx.

The clinical underestimation of the presence of massive tissue edema in the oral cavity represents an increased risk for the patient at the time of transport and during evaluation with a neck and chest CT exam with a contrast substance. There is a risk of producing an acute respiratory arrest that may go unnoticed. Oral or nasotracheal intubation should not be performed because of the risk of injuring the acutely obstructed airway. Tracheotomy or cricothyroidotomy under local anesthesia is recommended.

Deep neck infection (DNI) affects the fascial spaces of the neck, and can be fatal [10]. DNI may cause airway compromise, which is associated with serious morbidity, and even mortality. Protecting the airway is essential for managing DNI [40]. Tracheostomy is considered for DNI patients when intubation is hard to perform. However, the decision of whether to perform a tracheostomy usually depends on the physician’s clinical consideration. Artificial intelligence (AI) allows computers to perform tasks that normally require human intellect and cognitive processes [41]. Machine learning is a form of AI that allows predictions to be made based on information extracted from input data [42,43,44]. Such machine learning methods are referred to as deep learning (DL). However, to date, no DL model is available to help physicians to determine when to perform tracheostomy in cases of DNI, especially when there is no obvious sign of airway obstruction. Thus, our goal was to establish a DL model for predicting the need for tracheostomy in patients with DNI, based on their clinical and CT data. This would help clinicians to decide whether tracheostomy should be performed in cases of DNI, and may lead to improvements in critical care [41].

Infections of the retropharyngeal space present with systemic toxicity, fever, odynophagia, dysphagia, dyspnea, and neck stiffness. Oropharyngoscopic and videofibroscopic examination may show a bulging of the retropharyngeal region. The protrusion of the pharyngeal wall causes respiratory distress, compressing the supraglottic area. The extension of the infection into the danger space or the prevertebral space causes chest pain that may be due to pleural irritation, suggesting the involvement of the pleura or mediastinal spaces, and pain and stiffness localized to the neck. Involvement of the danger space gives rise to the most dreaded complication of retropharyngeal infection, acute bacterial mediastinitis, which may involve the posterior mediastinum through the danger space or the anterior mediastinum by spreading over the pretracheal fascia to penetrate the parietal pericardium. This infection has a fulminant evolution and a high mortality rate [4].

The infection may spread to the lateral pharyngeal space from the submandibular space via the styloglossus muscle, or may occur following a dental, retropharyngeal, peritonsillar, salivary gland, or local lymph node infection. It is necessary to determine the anatomy of the cervical region for the clinical assessment of the infection of this space. Infection of the anterior (prestilian) compartment of the lateral pharyngeal space presents with pain, fever, stiffness, and trismus in a toxic-appearing patient. Local examination shows the presence of pain in patients when the neck is turned to the side opposite to the infection, due to spasming of the sternocleidomastoid muscle. In contrast, infection of the posterior (retrostylian) compartment does not cause trismus or obvious tissue swelling. Involvement of the parotid gland (through a defect in the deep cervical fascia), however, is common, and leads to swelling of the parotid space. The main clinical findings are fever, odynophagia, and general toxic state. The presence of epiglottis edema can lead to dyspnea. Given the anatomical structures contained in the retrostylian space, the number of complications is increased by erosion or thrombosis of the carotid artery and thrombosis of the internal jugular vein [45,46].

Necrotizing fasciitis is the most dreaded form of cervicomediastinal suppuration, with fulminant onset caused by infection with anaerobic bacteria. At presentation, the patient may have a relatively good general condition in contrast to the pathogenesis of the disease. The onset of symptoms coincides with extensive infection with tissue necrosis. Severely affected patients show symptoms and signs of sepsis, which has a poor prognosis. If aggressive surgical treatment is not instituted early, the patient presents complications that increase mortality through the appearance of shock. Patients with necrotizing infections present arterial hypotension in 50% of cases, and acute renal failure, impairment of liver function, and acute respiratory distress in 10–30% of cases [28,46]. The patient initially presents with cervical swelling, pain upon palpation, edema, and erythema. The progression of the infection leads to the appearance of crepitations or emphysema at the level of the neck and upper chest, with the appearance of local ischemia and necrosis, clinical pallor, and necrosis of the skin. Necrotizing cervical fasciitis has a downward direction, with severe evolution and the appearance of mediastinitis. Mortality can be reduced by early diagnosis and therapeutic conduct.

Acute mediastinitis after traumatic perforations at the crico-pharyngeal level (upper esophageal sphincter) is usually detected early. The main symptoms, according to their frequency, are the following: dysphagia (80.6%), retrosternal pain with interscapular radiation (56.5%), nausea and vomiting (50%), fever and chills (38.7%), dyspnea (19.4%), and confusion (15.5%) [45]. Clinical examination usually reveals tachypnea, tachycardia, edema of the face and neck, subcutaneous emphysema located at the level of the chest or basi-cervical area, and Hamman’s sign with crackles on heart auscultation [45,46,47].

Comorbidities (diabetes mellitus, HIV infection, cancer, autoimmune diseases) must also be taken into account in developing the diagnostic and therapeutic management of patients with deep throat infection. Diabetes mellitus is the most common systemic disease associated with deep throat infection. Patients with comorbidities (immunocompromised) have a higher risk of developing fatal complications, even if the signs and symptoms are minor [35].

In patients with cervicomediastinal suppurations, the values of leukocytes, C-reactive protein, fibrinogen, ESR, procalcitonin, presepsin, hemoglobin, renal and hepatic function, and blood sugar are evaluated from admission and in dynamics. Follow-up of inflammatory markers after the initiation of treatment helps in decision-making in therapeutic management. In various studies, it is observed that the C-reactive protein value is significantly higher in patients with multiple abscesses in different cervical spaces [43]. In isolated abscesses, the number of leukocytes may remain normal. Blood analyses in patients with necrotizing fasciitis show metabolic acidosis and increased values of lactate and creatinine, with rapid evolution towards septic shock [43].

The bacteriological examination of the germs involved in infections of cervicomediastinal suppurations is carried out through microbiological tests to highlight the germs, as well as an antibiogram to establish the antibiotic treatment with the highest sensitivity. In recent years, there has been a significant increase in carriers of pathogenic staphylococci. The collected samples, after being tested via Gram staining, are cultured on two blood agar plates (one for aerobes and one for anaerobes), MacConkey agar, and Sabouraud agar, for 5 days at 37 °C [48].

The gold standard in the diagnosis and therapeutic management of DNM is dynamic CT. This method also identifies damage to the upper respiratory tract before the appearance of clinical symptoms (dyspnea, stridor). Computer tomography is the most used imaging modality in emergency situations. It can be used to establish the extension of the infection in the anatomical spaces adjacent to the initial necrotic process. Knowledge of radiological anatomy makes surgical approaches to deep neck infections accessible. Identifying the deep spaces of the neck involved in the infection allows the radiologist to evaluate the ways in which the infection spreads, and the probable causes of suppuration [48]. CT with a contrast substance is the first choice in emergency medical centers, being accessible, with fast image speed, and the ability to visualize the local extension of the infection. It should be noted that CT has a false positive rate of approximately 10% and a false negative rate of 13%. Retropharyngeal cellulitis can often have the CT appearance of an abscess, which leads to an increased rate of false positive results. The use of multiplane helical CT results in better resolution and a substantial reduction in scan acquisition time and display time, and this method is non-invasive. Multiplanar reconstructions via CT can be utilized to effectively distinguish fat from other tissues, and are superior to MRI in terms of evaluating bone and calcifications. Compared to MRI, CT has the advantages of being less susceptible to motion artifacts and having better temporal resolution [49]. Although CT scanning has an important role in the diagnosis of cervicomediastinal suppurations, it is not 100% predictive. Smith and colleagues [50] evaluated the positive predictive value of CT in deep throat infections. A total of 75% of surgically drained patients had a discrete pus collection that correlated with the CT findings; however, 25% did not. It was concluded that the decision for surgical drainage should be made based on clinical symptoms and signs, and that a negative exploration rate of 25% should be expected. Munoz and colleagues [51] compared CT with MRI in the evaluation of head and neck infections. Although MRI has been shown to be efficient in defining spaces and identifying the source of the infection, CT has been utilized to depict the presence of gas and calcifications [46].

Swelling of the neck that extends to the sternal notch may indicate the infectious involvement of the mediastinum, and a CT scan of the neck and chest should be performed in such cases [52,53]. The advantages of MRI are represented by the avoidance of exposure to radiation, the non-use of allergenic contrast substances, and a better evaluation of the limits of infection in soft tissues than is provided by CT [54]. In the event of vascular complications (thrombosis of the internal jugular vein, or aneurysm or rupture of the carotid artery), magnetic resonance angiography is recommended [37]. Unfortunately, its increased cost and longer scan time compared to CT are disadvantages that preclude its utility in most cases [50].

X-rays may also be helpful in certain cases of deep throat infections. Even if a chest X-ray can detect complications such as mediastinitis, pneumonia, and pleural effusion, CT evaluation is preferred, as it is clearly superior for detecting mediastinal cellulitis or abscesses [35]. In the case of deep neck infections with the presence of a deep abscess, CT can be supplemented with ultrasound, which is more precise in differentiating an abscess from cellulitis, and needle aspiration can be attempted [35,39].

## 5. Treatment of Deep Neck Infections Associated with Descending Necrotizing Mediastinitis

The management of cervicomediastinal suppurations, which represent a medical-surgical emergency, requires the institution of treatment within the shortest possible time, because the complications that may occur put the patient’s life at risk. A multidisciplinary team is required to reduce mortality, with the initiation of empiric antibiotic treatment and the imposition of aggressive surgical treatment through the drainage and debridement of the involved tissues.

Acute airway obstruction is one of the most common and deadly complications of cervicomediastinal suppurations. This is most common in cases with the involvement of multiple cervical spaces; Ludwig’s angina; or retropharyngeal, parapharyngeal, or anterior visceral space abscesses [36,55]. Airway permeability is maintained by performing a tracheotomy under local anesthesia or via orotracheal intubation. Aggressive management of the airway is required when the tongue is moving upwards, or when there is edema of the respiratory tract or obstruction caused by the presence of an abscess. Clinical signs of respiratory distress (stridor and dyspnea) appear [39]. A study by Parhiscar et al. found that up to 75% of cases of Ludwig’s angina require securing of the airway, and the authors recommended elective tracheotomy as a preventive measure in these patients [6]. In DNI with DNM, the evaluation and monitoring of the respiratory tract is the first step in therapeutic management, even before CT evaluation, and its parameters should be monitored in the first 24 h after the surgical intervention [38].

Orotracheal intubation can be attempted before tracheotomy in most patients who have cervical suppuration. Communication between the surgeon and the anesthesiologist is imperative, given the airway challenges in deep throat infections: the presence of trismus; throat swelling; and mass effect and edema of the tongue, pharynx, and larynx. If the vocal cords can be visualized in indirect laryngoscopy, orotracheal intubation is safe [56]. The use of a laryngoscope could rupture an abscess with a lateral pharyngeal or retropharyngeal location, resulting in aspiration of purulent material, with subsequent pulmonary complications.

To secure the airway, orotracheal intubation or tracheotomy can be performed under local anesthesia. Each has advantages and disadvantages that must be carefully considered when choosing the safest and most appropriate procedure. Some authors argue that the routine use of intravenous steroidal anti-inflammatory drugs in patients with impending airway obstruction can minimize swelling and eliminate the need for a tracheotomy [37,57]. However, NSAIDs have hyperglycemic effects, and should be avoided in patients who have diabetes or hypoglycemia [39]. The advantages of orotracheal intubation are rapid airway control and avoidance of the risks associated with a surgical procedure, and its disadvantages include accidental extubation, which is the most common complication of intubation. Extubation, with a loss of the airway following worsening of advanced laryngeal edema, and a subsequent inability to reintubate, is a source of avoidable mortality [49,50].

Some authors consider tracheotomy under local anesthesia as a safe procedure to ensure airway patency and as the first step in the surgical management of cervicomediastinal suppurations [57]. It is recommended in cases of airway obstruction by extrinsic compression or severe local edema, in the presence of accentuated trismus, or when orotracheal intubation cannot be performed [35]. Tracheotomy should be avoided as much as possible when the infection includes the anterior cervical or pretracheal space, as this procedure increases the risk of anterior mediastinitis [39]. Advantages of tracheotomy include securing the airways, allowing suction of tracheobronchial secretions so that patients spend less time in the ICU, reduced hospitalization, and lower mortality. Disadvantages of tracheotomy include tracheal stenosis of approximately 30% in any tracheostomy; surgical risks such as bleeding, esophageal injury, and pneumothorax [50]; pus aspiration; airway loss; arterial dissection; and exitus [57]. Cricothyrotomy can provide urgent airway access when complete airway loss requires immediate surgical intervention. Passage of a cricothyrotomy tube can cause trauma to the posterior wall of the trachea, and subglottic stenosis is a potential complication. Cricothyrotomy should be converted to a standard tracheotomy within 24 to 48 h [39].

### 5.1. Medical Treatment

Antibiotic treatment is instituted within the first hours after the appearance of clinical signs of infection in the cervical region. The chosen therapeutic scheme is based on statistical probability criteria (presumptive clinical and bacteriological diagnosis); an antibiotic or a combination of broad-spectrum antibiotics is selected to cover both anaerobic and aerobic bacteria. In recent years, we have witnessed a dynamic of the microbial spectrum, as well as the presence of germs that are resistant to classic antibiotic therapy (staphylococci resistant to beta-lactam antibiotics). Empiric antibiotic therapy must be applied to every patient who has cervicomediastinal suppuration initially, until culture and sensitivity results are available. Empiric therapy should be effective against the aerobic and anaerobic bacteria that are commonly involved, and when culture results and susceptibility testing are available, they allow for an appropriate antibiotic therapy to be tailored. Infections with an oropharyngeal starting point require empirical treatment to target beta-lactamase-producing streptococcal and staphylococcal organisms, as well as anaerobes [58].

There are studies that recommend, for optimal empiric coverage, the use of penicillin in combination with a beta-lactamase inhibitor (such as amoxicillin or ticarcillin with clavulanic acid), or a beta-lactamase-resistant antibiotic (such as cefoxitin, cefuroxime, imipenem, or meropenem) in combination with a drug that is highly effective against most anaerobes (clindamycin or metronidazole) [35,36,37,51]. Vancomycin should be considered for empiric therapy in patients with MRSA infection [14,34], and in patients who have neutropenia or immune dysfunction [59]. The addition of gentamicin for Gram-negative bacteria is effective for coverage against K. pneumoniae, which is resistant to clindamycin and is recommended for diabetic patients [37,45]. In patients at risk of renal disease, administration of aminoglycosides requires the monitoring of renal function [39]. In some studies, the most commonly used empiric antibiotic therapy option is a combination of ceftriaxone and metronidazole or replacement of metronidazole with clindamycin or amoxicillin/clavulanic acid [60,61]. The especially frequent association of the etiology of cervicomediastinal suppurations with dental anaerobic bacteria requires the use of clindamycin or metronidazole. However, there are studies showing that clindamycin can no longer be considered a first-line antibiotic in cervicomediastinal suppurations, as current rates of resistance among Bacteroides fragilis strains range from 20% to 50% or more worldwide [62].

Targeted antibiotic treatment is instituted after the obtainment of the microbiological results enabling the establishment of the germs involved in the infection, as well as the result of the antibiogram (48–72 h after the collection of the samples), and the duration of administration is decided according to the serial collections of secretions from the operative wound and the microbiological results. Although surgical drainage is the classic approach to any deep neck infection with suspected abscess formation [6,38,63], recent studies have begun to demonstrate that selected cases of an uncomplicated deep neck abscess or cellulitis can be effectively treated with antibiotics and close monitoring, without the need for surgery or drainage. Concomitant medical treatment for associated comorbidities such as diabetes mellitus may improve the overall immune status of a patient with deep throat infection [39].

Treatment with steroidal anti-inflammatory and non-steroidal anti-inflammatory drugs can be administered in increased doses for short periods (of 3–5 days). In the case of fungal infections, medicinal treatment is administered according to a fungigram, and anti-inflammatory treatment is discontinued. Thus, the need for surgical intervention is reduced in some patients [37,64]. These conservative treatment indications do not apply to diabetic patients, whose evolution is different [36].

Medical treatment includes symptomatic treatment, hydro-volemic rebalancing, and combating septic shock (oxygen therapy, vasopressors, cardiac tonics, nasogastric tube feeding). In frequent cases, collaboration between the ENT doctor, the intensive care doctor, the infectious disease doctor, and the laboratory doctor in necessary in order to establish the therapeutic scheme. This collaboration through targeted therapy leads to a decrease in the occurrence of complications and a decrease in mortality rate.

### 5.2. Surgical Treatment

Deep neck infection (cervicomediastinal suppuration) remains a frequent condition that can be life-threatening. Knowledge of the complex spaces of the neck and how they communicate is necessary for proper surgical management. The location of the infection can provide information regarding the source of the infection and the likely microorganisms that need to be covered when empiric therapy is instituted [46]. Infections of the deep cervical spaces of the neck, in the case of their extension and propagation to several spaces along the cervical fascia, require aggressive surgical treatment through the drainage and debridement of the tissues involved [65]. Knowledge of the anatomy of and relationships between the deep spaces of the neck is necessary for the adequate drainage of the neck during incision and debridement [58]. The drainage of these spaces, which is visualized in CT examination, is performed through incisions on the edge of the bilateral sternocleidomastoid muscle and the vascular bed, and the internal face of the sternocleidomastoid muscle is approached. If the peripharyngeal or perilaryngeal muscles are involved, the spaces are drained with gentle, sometimes digital, removal in this area.

There is a debate in the literature regarding non-surgical management or minimally invasive intervention (aspiration and drainage by radiological guidance). Percutaneous echo-guided drainage has been concluded to be an effective treatment for well-defined unilocular abscesses [47]. In recent studies, in the case of well-defined unilocular abscesses, in patients who do not require securing of the airway, minimally invasive techniques have been practiced. The drainage of percutaneously accessible abscesses is conducted via ultrasound guidance, and the approach to deep abscesses is conducted through CT guidance and needle aspiration. Needle aspiration and catheter placement offer the advantages of a small entry point, rapid healing time, no scarring, and a lower risk of contamination of surrounding deep spaces while draining pus [66]. Conservative management of a deep neck abscess with intravenous antibiotics, with or without needle aspiration, is used more often in the pediatric population. In a recent study by Cramer et al., adult patients had a significantly higher risk of morbidity and mortality if surgery was delayed, with a 2.4-fold increase in risk if surgery occurred after day 2 of hospitalization [58].

The external cervical approach is the most frequently used for DNI drainage, especially when several cervical spaces are involved, with the most important and difficult to access space being the retropharyngeal space. In the case of DNI with DNM, wide detachment and the total debridement of the necrotic tissue are necessary for a favorable evolution [35,39].

Surgery remains the main treatment for more complicated or severe cases of cervical suppuration. Indications for surgery include airway compromise, critical condition, septicemia, complications, downstream infection, diabetes mellitus, or situations in which no clinical improvement is noted within 48 h of the initiation of parenteral antibiotics [6,36,37,55]. For abscesses greater than 3 cm in diameter involving the prevertebral and carotid spaces or involving more than two spaces, surgical drainage is recommended [33].

The decision to perform a tracheotomy is made at the time of establishing the management of the case of cervical suppuration. Affected areas requiring extensive debridement should be kept open with antimicrobial dressings to allow frequent inspection and debridement. In cases where the infections extend downward, it is observed whether the infection spreads below the carina; at that moment, transthoracic drainage is necessary, involving a mixed team with a thoracic surgeon [54]. After the wound and tissues have been debrided and thoroughly examined, the region is irrigated with copious amounts of sterile saline until purulent discharge is no longer observed. Penrose drains can sometimes be placed locally, and inserted into each incision and sutured to the skin to prevent dislodgement. An absorbent dressing is applied to prevent maceration of the surrounding skin.

In cases of cellulitis involving several important fascial spaces, surgical treatment is recommended, just as in the case of abscesses, for which a wide incision and drainage are practiced, which leads to faster resolution by changing the local environment to one that is more favorable for the administration of antibiotics and for the activation of local defense mechanisms. The preference of many authors when dealing with infections that have progressed to the neck is to create an incision large enough to allow bimanual digital inspection and palpation of the spaces when anatomically possible. This inspection helps to ensure the adequate drainage of all involved areas, and facilitates wound irrigation [47]. The inflammation and maceration of the tissues involved in DNI can also be controlled digitally within the spaces using anatomical landmarks, resulting in efficient drainage, reduction in unexplored areas, faster recovery of patients, and a decrease in hospitalization time. Some authors recommend using separate incisions for tracheotomy and neck drainage procedures, to avoid the spread of infection to the mediastinum [35].

The management of cervical suppurations, especially in cases presenting complications from presentation or in evolution, as well as in patients with comorbidities, requires the interdisciplinary collaboration of ENT and OMF professionals, infectious diseases doctors, pharmacologists, pulmonologists, medical radiology imaging professionals, thoracic surgeons, and intensive care staff. There is a broad consensus on the need for multidisciplinary treatment of severe deep throat infections. Most authors agree on the need for the surgical drainage of accessed formations, which, in addition to having immense therapeutic significance, provide microbiological samples for bacteriological examination [13,15,67]. Several questions regarding surgical therapy are still under debate, namely, the suitable interval between the day of hospitalization and the first intervention [67], the frequency of surgical debridement [13], and the frequency with which drained fascial spaces should be washed [15,16,18,68].

Laryngeal infections through contiguity and lymphatic drainage also affect the thyroid gland, which requires surgery to drain the peritracheal spaces and the thyroid gland area, and in the case of a specified extension in the upper mediastinum, digital removal is performed.

The general consensus regarding the airway approach in Ludwig’s angina is to perform a tracheostomy, due to the increased risk of obstruction, and an oral intubation maneuver can exacerbate this obstruction [69]. Some authors state that once the airways have been protected, antibiotic therapy is curative in approximately 50% of cases, and that these patients do not need additional surgical therapy. However, surgical therapy is indicated if the CT examination of the neck shows the presence of purulent collections. When the infection involves only the sublingual space, intraoral drainage incisions are used, and when the infection involves several cervical spaces, an external submandibular approach is performed, with the dissection of the mylohyoid muscle. The literature states that approximately 20% of these cases require tooth extraction [70].

The management of lateral pharyngeal space infections is similar to that for Ludwig’s angina, but airway compromise is much less common. Open surgical drainage and debridement are recommended to prevent the spread of the infection in the retropharyngeal space, but also the erosion of the carotid artery or the internal jugular vein [70,71]. Some authors argue that drainage of the parapharyngeal space abscess is best approached through an incision at the level of the hyoid bone, which allows access to the posterior belly of the digastric muscle, which is retracted superiorly, and the dissection here allows access to the parapharyngeal space and drainage of the abscess [58].

In retropharyngeal infections, the surgical technique used includes a transcervical approach to control upper mediastinal infection. In patients with an infection that has descended below the level of T4, a mixed ENT–thoracic surgery is required for the drainage of pleural and pericardial collections. Complications of infection of the retropharyngeal spaces are common, including extension to adjacent sites, such as the meninges, epiglottic area, lung, trachea, or pharynx. Mortality in the pre-antibiotic era was greater than 50%, and remains high today, ranging from 25% to 42% [69]. The drainage of the retropharyngeal abscess should be planned at an adequate level to reach the abscess site and cavity. The surgeon must bear in mind that abscesses in this space may extend into the mediastinum, and an incision one or two widths above the clavicle may be very advantageous. This is often helpful in cases where there is also thyroid gland involvement. Deep and medial dissection to the carotid sheath provides access to the retropharyngeal space [58].

Failure to drain deep neck abscesses prolongs hospitalization and leads to complications. DNIs with DNM are fulminant, and must be treated urgently, as they are able to cause rapid changes in the patient’s condition within a few hours, with the appearance of sepsis and shock [58]. In DNI with DNM, there are no treatment guidelines, but the aggressiveness of the disease and increased mortality require the need for a multidisciplinary team. The multidisciplinary team should include an otolaryngologist, a maxillofacial surgeon, a thoracic surgeon, a radiologist, an infectious disease physician, a radiologist, an intensive care specialist, and an anesthesiologist.

The initial treatment of DNM includes securing the airways, surgical management via cleaning up infectious foci (pharyngeal or dental), and the drainage and debridement of neck and mediastinal tissues. Early postoperative complications through the occurrence of pleuro-pulmonary infections until the onset of sepsis require intensive postoperative care, with the simultaneous management of comorbidities.

In various studies, adequate mediastinal drainage remains controversial. Several reports have established that transcervical mediastinal drainage is sufficient for DNM drainage limited to the upper part of the mediastinum (Endo type I). In many studies, some surgeons recommend sternotomy on the midline, or a subxiphoid approach for the drainage of the lower part of the anterior mediastinum (Endo type II A), and they recommend posterolateral thoracotomy for the drainage of the lower part of the posterior mediastinum (Endo type II B).

In patients with sepsis, these aggressive approaches would increase morbidity, length of hospitalization, and mortality. To avoid complications, the VATS surgical approach is recommended for the drainage and debridement of infections under the carina. The advantages of this procedure are a better visualization of the entire thoracic cavity than the subxiphoid approach, a significantly smaller local trauma, and lack of complications of osteomyelitis and sternum dehiscence. In cases with repeated reinterventions, the VATS procedure is less invasive and better tolerated by the patient. The main disadvantage of this procedure compared to the sternal approach remains the high risk of pleural and pulmonary contamination due to the need to change the operative position [72].

Dissector expertise is necessary in order for pathologists to identify the structures that are involved in the deep neck infections [73]. Autopsy is important to identify dangerous lesions in DNI and DNM. CT or MRI can be conducted post-mortem to evaluate the role of instrumental analysis [73]. Post-mortem assessment of the causes of death can be challenging, and may have possible medicolegal consequences [74].

**Figure 4:**
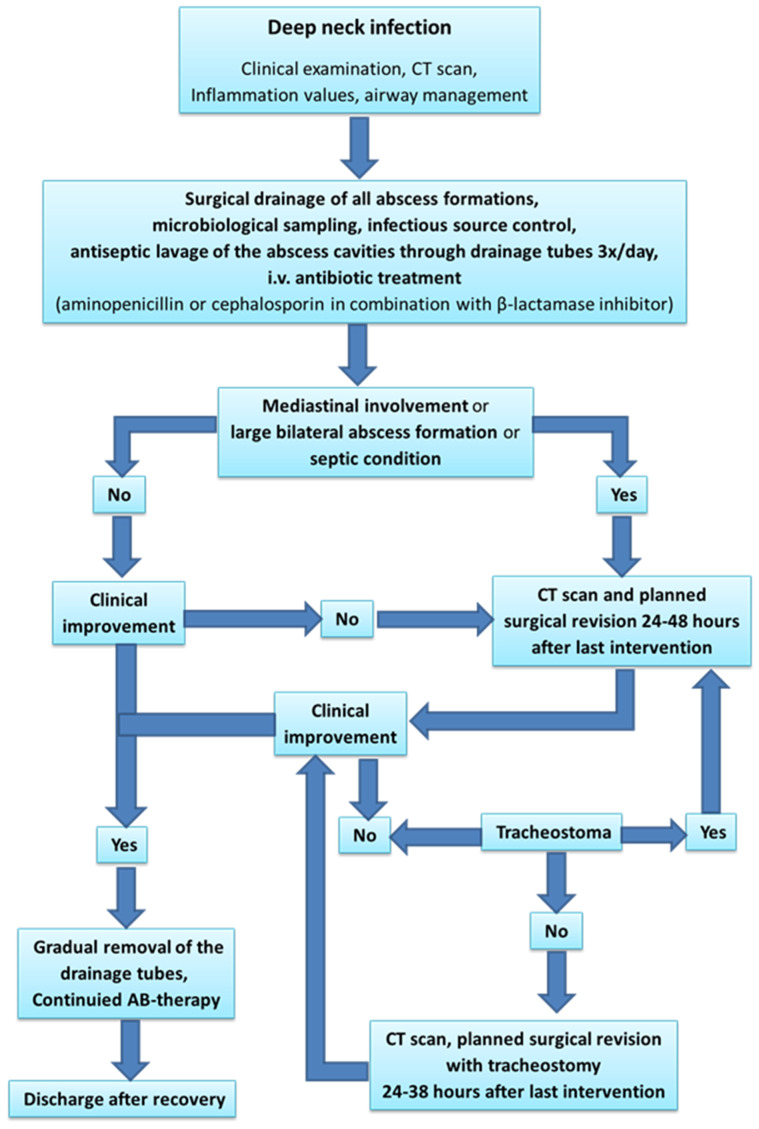
Management of deep neck infections.

A specific general algorithm for better outcomes in the management of deep neck infections has been proposed by T. Gherke et al. in the *European Journal of Otorhinolaryngology*, as shown in Figure 4 [19].

## 6. Limitations and Future Perspectives

One limitation to the present scoping review could be the exclusion of articles for which the full text was not freely available online. In the present context of publishing, we believe that all major breakthroughs should be made available through open access. However, we encourage fellow scientists to send published or unpublished data on the subject of the present review to the corresponding author. We hope that the current scoping review will be the cornerstone for the design of future studies on deep neck infections and necrotizing descending fasciitis. There is an increasing amount of data regarding these types of pathology in Romania. A recent study underlined the need for developing tertiary unit teams with a multidisciplinary treatment approach [75]. Table 2 summarizes the current level of evidence regarding all the steps in the successful management of deep neck infections.

## 7. Conclusions

Most lesions have an otorhinolaryngological (ORL) and oromaxillofacial (OMF) starting point. The infection spreads through contiguity, through the lymphatic or hematogenous route, following contamination of mucosa mainly of dental, buccal, pharyngeal, laryngeal, submaxillary, or parotid glandular inflammatory origin, but also of iatrogenic origin, following endoscopic maneuvers or orotracheal intubations. However, initial evaluation of the airway is a priority, and any signs of respiratory distress or impending airway compromise should be immediately and aggressively managed. A course of wide-spectrum antibiotics should be started empirically and changed according to bacteriological culture and sensitivity reports.

In the last two decades, progress in diagnostic techniques, surgical treatment, and antibiotics, as well as improvement of anesthesia and intensive care protocols, have led to a significant decrease in mortality rates in patients with DNI and DNM. Early and aggressive treatment instituted based on CT results can stop the evolution of the infection towards DNM. Dynamic CT follow-up 48–72 h after the primary surgical intervention may indicate the need for a reintervention to drain a residual abscess, or excision of the necrotic tissue.

The use of a multidisciplinary team in the management of DNI associated with DNM can lead to a decrease in serious complications, sepsis, and mortality.

## Figures and Tables

**Table 1:** A literature review of the mediastinal involvement of etiological factors in DNI.

	Misthos et al., 2007 [14]	Roccia et al., 2007 [13]	Ridder et al., 2010 [15]	Kocher et al., 2012 [16]	Celakovsky et al., 2014 [17]	Kimura et al., 2020 [18]	Gehrke et al., 2022 [19]	Ho et al., 2022 [20]
Odontogenic	63%	39.1%	11.1%	5.9%	40%	5%	15.56%	38%
Pharyngo-tonsillar	37%	60.9%	46.7%	82.3%	33.5%	33%	55.56%	19.1%
Retropharyngeal abscess	-	-	-	-	-	5%	-	19.1%
Other	-	-	42%	-	6.5%	18%	28.88%	-
Unknown	-	-	-	11.8%	20%	39%	-	23.8%

**Table 2:** The current levels of evidence for each step of the current management protocol of deep neck infections associated with descending necrotizing mediastinitis.

Step of Current Management Protocol	Level of Evidence
Etiopathology	Level 1 ^(1)^
Clinical diagnosis	Level 2 ^(2)^
Paraclinical diagnosis	Level 1 ^(3)^
Airway management	Level 2 ^(4)^
Medical treatment	Level 1 ^(5)^
Surgical treatment	Level 1 ^(6)^

^(1)^ The highest level is for a metanalysis regarding the association between deep neck infections and diabetes. ^(2)^ The highest level is for a cohort study regarding clinical versus computed tomography evaluation in the diagnosis and management of deep neck infections. ^(3)^ The highest level is for a metanalysis regarding the association between ultrasonography-guided drainage versus surgical drainage for deep neck infections. ^(4)^ The highest level is for a cohort study regarding a clinical risk scale to predict the requirement of advanced airway management for deep neck infections. ^(5)^ The highest level is for a metanalysis regarding antibiotic therapy for pediatric deep neck infections. ^(6)^ The highest level is for a metanalysis regarding ultrasound-guided puncture drainage versus surgical incision drainage for infections.
